# Structural variant landscape provides insights into genome organisation and domestication in European seabass

**DOI:** 10.1186/s12915-025-02404-7

**Published:** 2025-10-01

**Authors:** Zexin Jiao, Robert Mukiibi, Manu Kumar Gundappa, Massimiliano Babbucci, James G. D. Prendergast, Diego Robledo, Luca Bargelloni, Daniel J. Macqueen

**Affiliations:** 1 The Roslin Institute and Royal (Dick) School of Veterinary Studies, The University of Edinburgh, Midlothian, UK; 2 Department of Animal Health Behaviour and Welfare, Harper Adams University, Edgmond, Newport, TF10 8NB England; 3 Present Address: Animal Breeding and Genomics, Wageningen University and Research, P.O. Box 338, Wageningen, 6700 AH The Netherlands; 4 Department of Comparative Biomedicine and Food Science (BCA), University of Padova, Legnaro, PD Italy; 5 Department of Zoology, Genetics and Physical Anthropology, Universidade de Santiago de Compostela, 15706 Santiago de Compostela, Spain

**Keywords:** Structural variants, European seabass, Whole-genome sequencing, Domestication

## Abstract

**Background:**

Structural variants (SVs) are genetic polymorphisms including deletions, insertions, inversions, and duplications, with potential to influence traits through impacts on gene function and expression. SVs have not been widely utilized in genetic analysis, owing to the challenge of their accurate detection and genotyping. Addressing this general issue, and the broader demand to understand their role in commercially important taxa, we report a comprehensive analysis of SVs in the genome of European seabass (*Dicentrarchus labrax*), the most commercially important fish in the Mediterranean region.

**Results:**

Using whole genome sequencing from a farmed population (*n* = 90 samples), 21,428 SVs were identified using a comprehensive detection and genotyping strategy involving manual curation of every variant. This high-confidence SV atlas was annotated to predict impacts on genes and evolutionarily conserved sequences. We explored the overlap between SVs and repeats, identified heterogeneity in SV density across the genome, and tested if the coding genes disrupted by SVs are enriched for specific biological processes or conserved protein domains. SVs impacting evolutionarily conserved genomic regions were enriched in genes with nervous system and developmental functions. Finally, we performed a comparative analysis incorporating 38,408 high-confidence SVs identified independently for three wild populations (*n* = 80 samples) using identical methods. An analysis of 41,336 SVs merged across the two datasets provides insights into genes and biological functions targeted during aquaculture domestication, with evidence of shifts in allele frequency for SVs located within or near genes controlling behaviour, enriched for forebrain and synaptic functions, and specifically expressed in the brain.

**Conclusions:**

This study sheds light on the global organisation of SVs across the European seabass genome, revealing a potential role in aquaculture domestication. The reported datasets provide a novel, high-quality reference for future genetic investigations of both farmed and wild European seabass.

**Supplementary Information:**

The online version contains supplementary material available at 10.1186/s12915-025-02404-7.

## Background

Structural variants (SVs) include a range of genetic polymorphisms, often defined as being larger than 50 bp in length, including deletions, insertions, inversions, duplications, and translocations [1–3]. SVs have larger potential to influence gene functions and expression than single nucleotide polymorphisms (SNPs) and indels (i.e. small insertions and deletions) and are widely implicated in human genetic disorders and diseases [3–8], with many studies demonstrating their impacts on both genes and regulatory sequences [9–15].

Compared to SNPs, SVs are underrepresented in population and functional genetic studies, primarily due to the additional challenge and cost associated with their accurate detection and genotyping [16]. Nevertheless, advances in sequencing and bioinformatics have enabled comprehensive analysis of SVs in many species, including non-model taxa [4, 17, 18]. There is a growing interest in understanding the role of SVs in shaping genome and trait architecture in aquatic species of commercial and ecological importance, for example: Atlantic salmon (*Salmo salar*) [19, 20], rainbow trout (*Oncorhynchus mykiss*) [21], lake whitefish (*Coregonus clupeaformis*) [22], Australasian snapper (*Chrysophrys auratus*) [23] and Pacific oyster (*Crassostrea gigas*) [24].

The European seabass (*Dicentrarchus labrax*) is a key aquaculture species in Europe, representing the first marine teleost cultured commercially after salmonids [25], with modern production levels reaching 244,000 tonnes [26]. Studies of farmed and wild populations have benefited from the availability of reference genome sequences alongside other key genomic resources (reviewed in Vandeputte et al. 2019) [25]. This includes SNP marker panels applied to selective breeding, targeting improvement in traits essential for productivity and fish welfare, achieved through quantitative trait loci (QTL) discovery, genome-wide association studies, and genomic prediction [27–32]. A recent study highlighted the value of combining genome-wide SNPs with functional genomics, to detect candidate causal variants for resistance to one of the key viral pathogens affecting European seabass aquaculture [33].

Despite the importance of genomics in European seabass, the landscape of SVs in the genome of this species remains uncharacterised. To tackle this resource and knowledge gap, this study aimed to establish and investigate a high-quality landscape of SVs mapped to the latest European seabass genome. A robust pipeline was established to detect and genotype high-confidence SVs, which were annotated to predict impacts on genome architecture and function. To explore the potential role of SVs in domestication, we performed comparative genetic analyses of SVs called using whole genome sequencing data from both farmed and wild European seabass populations. Our findings and the resources shared through this work have valuable applications in future studies investigating the genetic basis for traits of commercial, ecological and evolutionary importance in European seabass.

## Results and Discussion

### SV landscape in farmed European seabass

**Fig. 1:**
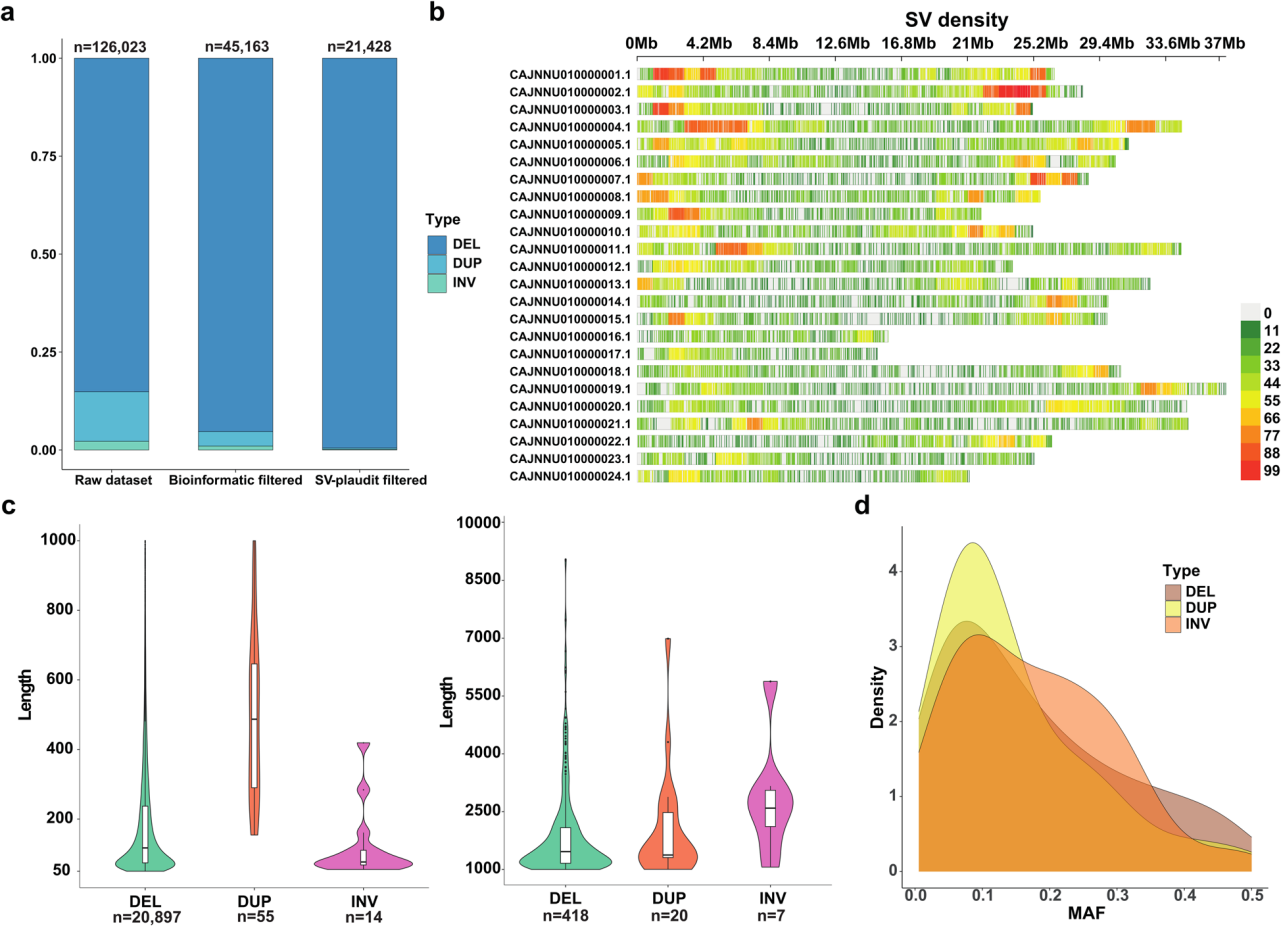
High-confidence SVs mapped across the European seabass genome. The data represents 21,248 SVs from a farmed population of *n* = 90 individuals. **a** SV counts before and after filtering steps. **b** SV density per 1 Mb intervals along different chromosomes. **c** Violin plot for deletions (DEL), duplications (DUP) and inversions (INV), split into two length ranges: 50 to 1,000 bp and 1,000 to 10,000 bp. **d** Minor allele frequency (MAF) density distribution plot for each SV class

126,023 SVs were initially identified from *n* = 90 whole genome sequencing (WGS) samples representing a farmed group of European seabass. After filtering of raw SV calls, 45,163 unique SVs were identified, representing 43,057 deletions, 1,654 duplications and 452 inversions (Fig. 1a). Using SV-plaudit, this dataset was reduced to 21,428 high-confidence SVs, representing 21,320 deletions, 75 duplications, and 33 inversions (Fig. 1a). Information about SV size, genomic locations, allele frequencies and SnpEff annotations are provided in Additional file 1: Table 1. We further generated a table of putative orthologous genes for every European seabass gene to facilitate subsequent data interpretations (Additional file 2: Table 2).

Across the genome, one SV was detected per 31,394 bp on average, with notable variation in SV density within and across chromosomes (Fig. 1b; Additional file 3: Table 3). The genomic location of regions showing the highest (> 80 SVs per Mb) and lowest (< 10 SVs per Mb) density of SVs are provided in Additional file 4: Table 4. The total genomic length of SVs was 6,510,004 bp, representing 0.94% of the reference genome length. The cumulative length of deletions, duplications and inversions was 4,735,175 bp, 67,711 bp and 1,707,118 bp, respectively. Around 90% of the SVs were < 1.5 kb, with average/median lengths of 241/121 bp (Fig. 1c). Deletions, duplications, and inversions showed average/median lengths of 223/120 bp, 904/619 bp, and 51,732/2,456 bp, respectively. The largest deletion, duplication and inversion was 32,027 bp, 6,987 bp and 402,664 bp, respectively, while 16 (0.08%), 1 (1.3%), and 13 (39%) variants from each SV category exceeded 5,000 bp (Additional file 1: Table 1). Three very large inversions (each ~ 400,000 bp) were co-located on CAJNNU010000016, showing > 95% overlap (Additional file 1: Table 1). These inversions have slightly different breakpoints and likely represent the same or closely related variants validated independently using SV-plaudit. The minor allele frequency (MAF) distribution of different SV types showed a peak of around 0.1 in each case (Fig. 1d) and 4,026 SVs exhibited MAFs < 0.05. The number and size distribution of SVs was similar to that observed in previous studies of salmonids using a similar pipeline [19, 21].

### SV annotation and functional impacts

**Fig. 2:**
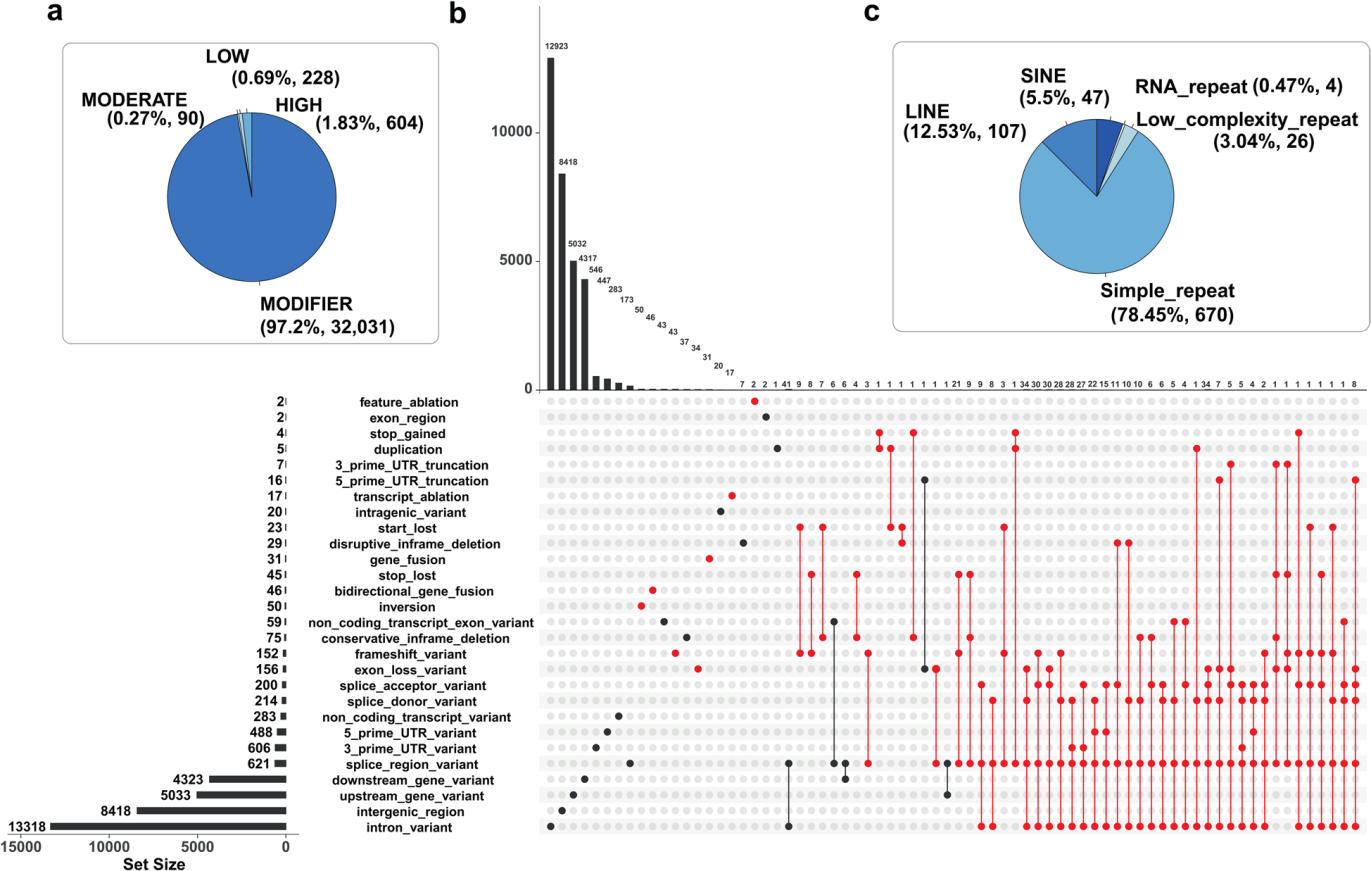
Annotation of SVs in the European seabass genome. The data represents 21,248 SVs from a farmed population of *n* = 90 individuals. **a** Proportions of SnpEff categories across the full dataset. **b** UpSet plot visualising the number of shared SnpEff annotations across different predicted impact categories. Each SV can have multiple SnpEff annotations, with varying impacts. High impact categories were indicated by red dots. **c** Number and type of repeat elements showing > 50% reciprocal overlap with SVs across the full dataset

SnpEff was used to annotate the 21,428 high-confidence SVs in the European seabass genome (Fig. 2a; Additional file 1: Table 1). Approximately 39%, 25% and 27% of all SVs were located within introns, intergenic regions, and 5,000 bp up/downstream of genes, respectively (Fig. 2b). A small fraction (3.19%) were located within untranslated regions (UTRs). Another small fraction (2.31%, 494 out of 21,428) were categorized as ‘high impact’ by SnpEff, representing 1.83% of all predicted SV impacts (Fig. 2a-b). An UpSet plot was used to visualize relationships between predicted impacts of all SVs, inclusive of high impact effects, which included exon losses, frameshift mutations, and gene fusions (Fig. 2b). A caveat is that the predicted SnpEff impacts depend on the accuracy of genome annotation—hence, while these results provide a valid global overview of SV effects, the impacts of SVs on specific genes warrants careful inspection of the quality of gene model predictions.

### Association between SVs and repeats

Past work using the same SV detection pipeline identified recently active repeat elements in the Atlantic salmon genome [19]. The same study identified a large number of deletion SVs of near identical length, representing closely-related sequences belonging to an evolutionarily young transposable element (TE) family specific to *Salmo* [19]. To explore the relationship between SVs and repetitive sequences and seek evidence for recently active TEs in the current study, we predicted repeat sequences and overlapped them with the 21,428 SVs from farmed seabass (Fig. 2c; Additional file 5: Table 5). 854 SVs (~ 4%) showed a > 50% reciprocal overlap with individual repeat elements, representing 670 simple repeats, 107 long interspersed nuclear elements (LINEs) and 47 short interspersed nuclear elements (SINEs) (Fig. 2c; Additional file 5: Table 5). Among these, only 73 and 6 reciprocal overlaps exceeded 90% and 99%, respectively.

Consequently, there is little support for a strong relationship between SVs and repeat sequences in the European seabass genome. Moreover, our SV dataset captures little evidence for recently active TEs, though it should be noted the SV detection pipeline deployed was not designed for this purpose. This finding sits in contrast to past studies in Atlantic salmon and rainbow trout, where a markedly larger overlap between high-confidence SVs (detected using a similar pipeline) and TEs was reported [19, 21]. For instance, Liu et al. (2021) showed that the repeat sequence content of rainbow trout SVs exceeded 85% [21]. Understanding the basis for such strikingly distinct relationships between SVs and repeat elements across teleost lineages will require further datasets, spanning more species.

### Are genes affected by high-impact SVs functionally enriched?

**Fig. 3:**
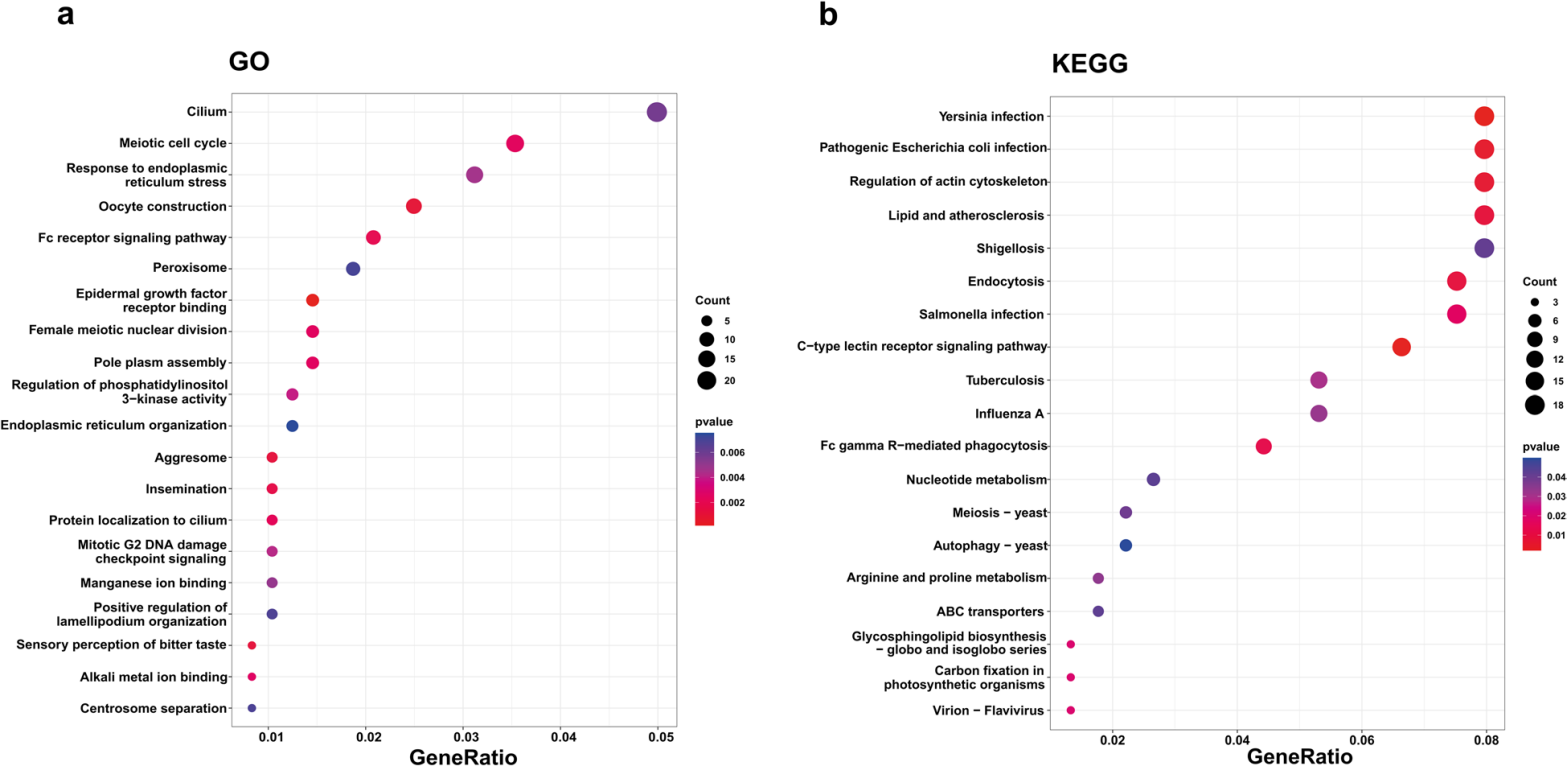
Enrichment tests for genes associated with high impact SVs in the European seabass genome. The analysis was performed using SVs from a farmed population of *n* = 90 individuals. Clusterprofiler plots are shown for **a** GO terms and **b** KEGG pathways. The x axes show the ratio of high impact SV affected genes in each term relative to the total number of SV related genes in that term. Dot sizes show the number of high impact SV affected genes per term, while color is the uncorrected *p* value. Full results are provided in Additional files Tables 6–8

Given the potential of SVs to disrupt gene functions, we tested whether specific biological processes or pathways are enriched among the 487 genes affected by high impact SVs according to SnpEff (Fig. 3; Additional file 6: Table 6 and Additional file 7: Table 7). We identified 233 enriched GO terms and 21 enriched KEGG pathways at uncorrected *p* < 0.05, but none remained significant after correcting for multiple comparisons (Fig. 3a, b). Terms enriched at *p* < 0.05 were explained by 285 unique genes (264 genes for GO; 90 genes for KEGG). This overall lack of strong enrichment suggests that SVs with predicted high impacts are not strongly biased towards particular biological functions. The most enriched KEGG pathways (all uncorrected *p* < 0.01) hinted at weak enrichment of high-impact SVs in immune genes associated with antibacterial functions, including “*Yersinia infection*”, “*C-type lectin receptor signaling pathway*” and “*Pathogenic Escherichia coli infection*”.

While not significant following correction, these enrichment tests provide a useful approach to group candidate genes affected by high-impact SVs based on shared functions (provided in Additional file 8: Table 8). For example, with respect to KEGG pathway “*C-type lectin receptor signaling pathway*”, we identified a 4,018 bp deletion (MAF: 0.09) that removes four out of five coding exons of a C-type lectin domain encoding gene (ENSDLAG00005002384), predicted to disable its coding potential (Additional file 8: Table 8). In the same pathway, a high impact 1,594 bp deletion (MAF: 0.12) is predicted to delete two exons encoding a large segment of another C-type lectin protein domain in a separate gene (ENSDLAG00005017891) predicted to represent a distant homolog of human *CLECF4*.

### Are genes affected by high impact SVs enriched for conserved protein domains?

As an alternative approach to investigate the impact of SVs on functions encoded within the European seabass genome, we asked if proteins encoded by genes affected by high impact SVs according to SnpEff are enriched for particular conserved functional domains. The proportion of InterPro domain annotations associated with the 487 genes affected by high impact SVs was compared to the remaining genes not affected by high impact SVs according to SnpEff (22,482 genes) using Fisher’s Exact tests (Additional file 9: Table 9). 27 InterPro domains showed enrichment at uncorrected *p* < 0.01, explained by 75 unique coding genes. After correcting for multiple comparisons, only 2 InterPro domains remained significantly enriched at adj. *p* < 0.05, explained by 4 unique coding genes (Additional file 9: Table 9). However, as Fisher’s Exact test is conservative, we accepted protein domains showing enrichment at uncorrected *p* < 0.01 to avoid false negatives.

The most enriched protein domains were “Transposase, L1” and “L1 transposable element, C-terminal domain” (*p* = 1.31e-05 and 3.73e-05, fold enrichment = 46.2 and 138.5, respectively), explained by four transposon coding genes (three shared by both InterPro domains). L1 transposons, also known as long interspersed nuclear element-1 (LINE-1), are extremely common in mammalian genomes, where they are the only known autonomously active retrotransposon, with almost all inactivated by mutations and SVs [34]. In the European seabass genome, each of the four genes encoding these LINE-1 associated domains (ENSDLAG00005000995, ENSDLAG00005026482, ENSDLAG00005030046 and ENSDLAG00005031661) have deletions disruptive to coding regions that are present at moderate to high allele frequencies (respective MAFs: 0.37, 0.37, 0.13, 0.35). Orthologs for several of these LINE-1 genes are predicted in other teleost species by Ensembl, indicating ancient retrotransposition dates. The enrichment of disruptive SVs in genes encoding L1 transposon domains, taken in light of the small number of genes containing these domains (8 for “L1 transposable element, C-terminal domain”; Additional file 9: Table 9) indicates an ongoing process of LINE-1 inactivation.

Another notable enriched domain was ‘5-Hydroxytryptamine 4 receptor’ (*p* = 4.49e-04, fold enrichment = infinite), which in humans is restricted to a single protein of the same name (abbreviated 5-HT4 receptor, encoded by *HTR4*), representing a serotonin receptor with essential roles in behaviour, cognition, and gastrointestinal motility and digestion [35]. In seabass, we identified two paralogs related to *HTR4* (ENSDLAG00005005527 and ENSDLAG00005011448), predicted by Ensembl to date back to early vertebrates. Both were affected by high impact SVs, representing the only two proteins in the genome harboring the 5-HT4 receptor domain (Additional file 9: Table 9). Another enriched domain was “Alpha-2-macroglobulin, TED domain” (*p* = 0.0063, fold enrichment = 23.1), explained by two genes (ENSDLAG00005004695 and ENSDLAG00005017199) encoding predicted homologs of Alpha-2-macroglobulin, a family that acts as broad-spectrum protease inhibitors with functions in humoral innate immunity [36]. A large deletion was identified in ENSDLAG00005004695 (MAF: 0.04) that ablates the last 14 exons of this gene (comprising 29 exons).

Other domains showing evidence for enrichment among coding genes affected by high impact SVs have diverse biological roles, including in neurotransmission, myelin formation, intracellular signaling and innate immunity, e.g., ‘Vav, PH domain’, ‘Guanine-nucleotide dissociation stimulator, CDC24, conserved site’, ‘Myelin and lymphocyte (MAL) protein’ and ‘Serine/threonine-protein kinase, active site’, as well as multiple NACHT associated domains. The enriched domain ‘Mitotic spindle checkpoint protein Bub1/Mad3’, is involved in the cell cycle, while ‘ABC transporter type 1’ and ‘Glycosyl transferase family 6’ domains have roles in metabolism, including membrane transport, glycosylation, and nucleotide processing. Domains related to transcriptional and translational regulation, including RNA-binding motifs and kinase domains (e.g., ‘TIAR RNA recognition motif’ and ‘MHCK/EF2 kinase’), highlight possible impacts on gene expression and protein synthesis. Finally, a set of domains such as ‘Somatomedin B’ and ‘Coagulation factor 5/8 C-terminal domain’ were implicated in extracellular matrix integrity and haemostasis.

Overall, this analysis underscores the broad and potentially pleiotropic effects of high-impact SVs on essential cellular and physiological processes in European seabass.

### Hundreds of SVs disrupt evolutionarily conserved regions

**Fig. 4:**
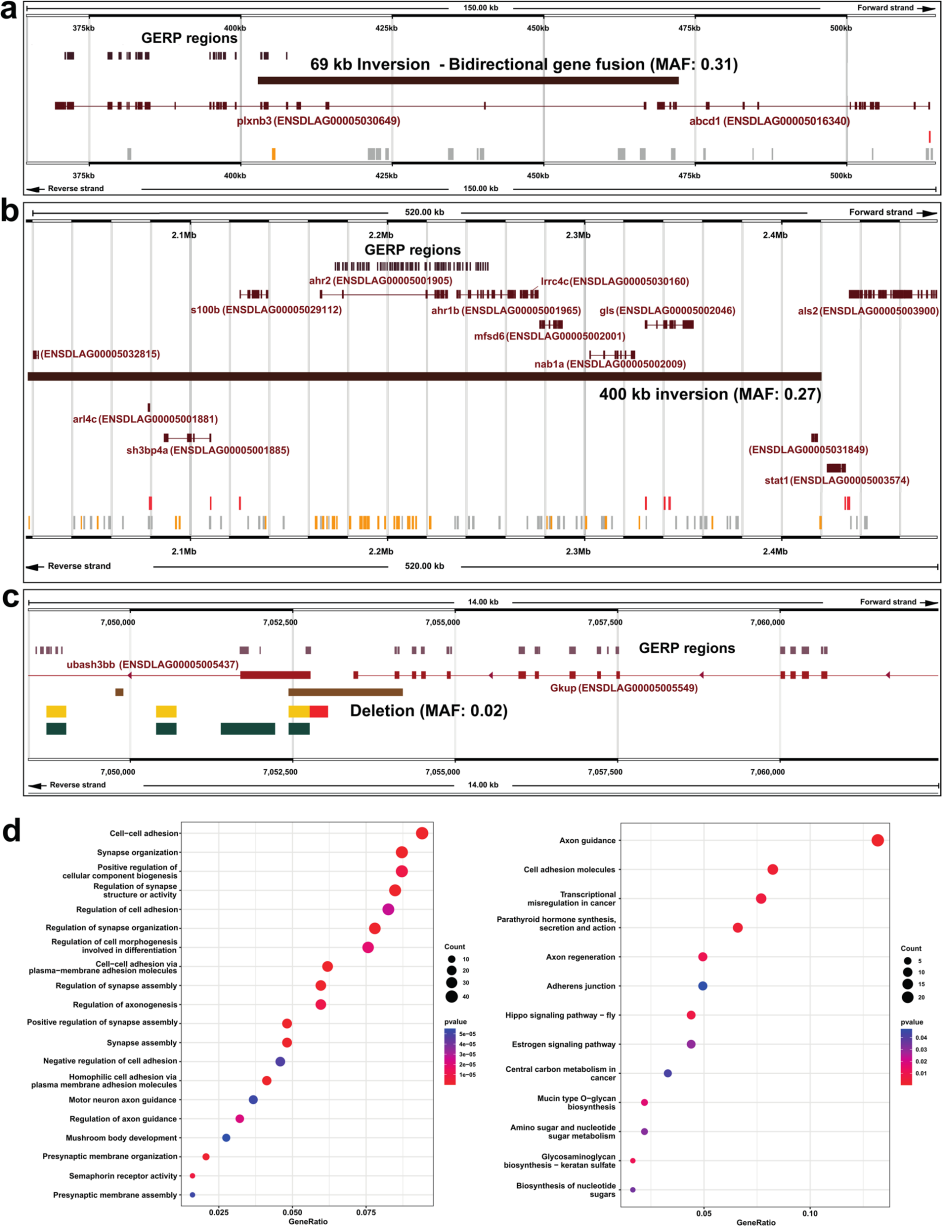
Overlap between SVs and evolutionarily conserved regions in the European seabass genome. The analysis was performed using SVs from a farmed population of *n* = 90 individuals. **a-c** Examples of SVs that affect GERP regions in genomic regions containing highly conserved genes discussed in the main text. **D** Clusterprofiler plots showing the top twenty most enriched GO terms (left) and KEGG pathway (right), using the full set of genes associated with SVs that overlap GERP regions. For KEGG, only the most enriched term “Axon guidance” was significant at adjusted *p* < 0.05. Full results are provided in Additional files Tables 12 and 13, including tests performed when SVs > 10,000 bp were excluded

As a third strategy to explore the impact of SVs on functional features in the European seabass genome, we focussed on genomic regions showing evidence of strong evolutionary constraint across species, a hallmark of purifying selection and functional importance. We overlapped the 21,248 high-confidence SVs from farmed seabass with evolutionarily constrained regions defined from a whole genome alignment of 65 actinopterygian species including European seabass (Fig. 4; Additional file 10: Table 10). These regions were defined using Genomic Evolutionary Rate Profiling, a statistical method that identifies aligned regions showing fewer substitutions than neutral expectations [37].

366 SVs (1.7%) overlapped 1,219 GERP-defined constrained regions (mean/standard deviation length: 61/77 bp) by at least 1 bp (mean/standard deviation of overlap: 47/58 bp). 17 of these 366 SVs (4.6%) were predicted by SnpEff to represent high impact variants, affecting 40 unique genes. This is approximately twice as high as the background rate of predicted high impact SVs (2.31%; 494 out of 21,428), which is significant (Chi-square test, *p* = 0.0078). Among these SVs, we identified a 69,461 bp inversion (MAF: 0.31) predicted to cause a bidirectional fusion between the first 7 exons of *plxnb3*, which overlaps a large cluster of constrained elements, and the last 3 exons of *abcd1* (Fig. 4a). *plxnb* and *abcd1* are highly conserved genes, with the European seabass genes predicted to share 51.3% and 67.4% protein-level identity with their human ortholog. In humans, *abcd1* encodes ALD, a protein that causes a disease affecting the nervous and adrenal system called adrenoleukodystrophy [38]. *plxnb* encodes Plexin 3B, a protein central to nervous system development, which acts as a receptor for semaphorin proteins [39]. A remarkably similar mutation involving a deletion spanning *abcd1* and *plxnb3* is associated with human adrenoleukodystrophy, a rare genetic disease affecting the nervous system and adrenal glands [40].

The three closely-related ~ 400 Kb inversions (MAFs: 0.17–0.27) noted earlier to represent the same variant, in addition to fully-encompassing ten protein coding genes, overlap a cluster of conserved GERP regions located throughout the *ahr2* and *ahr1b* genes (encoding aryl hydrocarbon receptor; AHR, family members), largely affecting non-coding intronic regions (Fig. 4b). Zebrafish orthologs of these genes have roles in xenobiotic metabolism, while *ahr2* was required for normal zebrafish behaviour [41]. A group of conserved genes affected by this inversion encode proteins with functions in the brain and nervous system: e.g. *s100b*, encoding a Ca2 + -binding protein up-regulated in mammalian astrocytes and a key biomarker for multiple nervous system disorders and traumas [42]; *mfsd6*, encoding a major facilitator superfamily domain-containing protein localised in the mouse brain, with a potential role in energy homeostasis [43]; *gls*, encoding glutaminase A, an enzyme essential for brain homeostasis that catalyses glutamine hydrolysis to produce glutamate—an important brain excitatory neurotransmitter – that has been associated with many neurodegenerative diseases [44]; *arl4c*, encoding a small GTPase important for mammalian brain development [45]; and *lrrc4c*, encoding a leucine-rich repeat protein with key roles in nervous system development [46]. Even genes proximal to this inversion have roles in the nervous system, including *stat1*, a master immune transcription factor with key roles in regulating brain inflammation and neural stem cell renewal in mice [47, 48], and *als2*, encoding a rho guanine nucleotide exchange factor called Alsin, which is highly expressed in neurons and the causal gene for characterised neural sclerosis diseases in humans including AMS [49].

Among the high-impact SVs overlapping conserved regions, we identified a 1,761 bp deletion (MAF: 0.02) predicted to ablate the last two coding exons of a gene called *gkup* (ENSDLAG00005005549; encoding glucuronokinase), which is a highly conserved single copy gene found across teleosts (e.g., ~ 80% protein-level identity between zebrafish and European seabass), with the deletion fully encompassing an exon representing a GERP region (Fig. 4c). This deletion also ablates the entire upstream region (including a promoter and enhancer predicted by Ensembl) and overlaps the first coding exon of *ubash3bb* (ENSDLAG00005005437; encoding ubiquitin-associated and SH3 domain-containing B), another highly conserved vertebrate gene (e.g., ~ 81% protein-level identity between zebrafish and European seabass). The remaining high-impact SVs overlapping conserved/GERP regions are characterised in Additional file 11: Table 11.

We also identified high-confidence SVs not in the high impact SnpEff subset, overlapping evolutionarily conserved non-coding regions, including intergenic regions, which may represent regulatory elements. For example, we identified a 3,409 bp deletion (location—CAJNNU010000002.1:10,854,297–10857705; MAF: 0.03) residing within a large intergenic region containing a large number of GERP elements, with the nearest genes ~ 92 Kb upstream (ENSDLAG00005013971) and ~ 33 Kb downstream (ENSDLAG00005013980). The region overlapped by this deletion is annotated by Ensembl as open chromatin, indicating its functional importance. As another example, we identified a 6,154 bp deletion (location—CAJNNU010000010.1:13,793,442–13,799,595; MAF: 0.07) embedded within a large intron of a gene (ENSDLAG00005024298) encoding SH2 domain containing 3Ca, a highly conserved (e.g., ~ 77% ID between seabass and zebrafish orthologs) adapter protein that influences cell signalling and adhesion. This deletion region includes a large number of GERP elements and four enhancers predicted by Ensembl to have activity in brain and embryos.

The above examples provide evidence that a subset of segregating SVs in European seabass genomes disrupt highly conserved vertebrate genes and non-coding elements, with impacts Likely affecting both protein function and gene regulation. To gain a global overview of the genes affected by these SVs, we performed GO and KEGG pathway enrichment tests on the 496 genes associated with all high-confidence SVs overlapping GERP regions (Fig. 4d; Additional file 12: Table 12 and Additional file 13: Table 13). As very large SVs may overlap a large number of genes, such as the above highlighted inversion example, we also performed the test excluding SVs defined as > 10,000 bp (*n* = 482 genes). The results were congruent in both tests, and highlighted enrichment of GO terms associated with brain and nervous system functions linked to synapse organisation, structure and assembly, semaphorin receptor activity, regulation of axon guidance, axonogenesis, neuron recognition, forebrain cell migration, ephrin receptor activity, among others (Additional file 12: Table 12). The KEGG analyses identified one term that remained significant after correction for multiple comparisons, “Axon Guidance”, consistent with the GO analysis (Additional file 13: Table 13). Beyond this clear signal of nervous system related functions, we also identified enrichment of terms associated with the development of other organs and systems, including the heart, the urogenital system, the exocrine system, as well as terms associated with embryonic development (Additional file 12: Table 12). These results indicate that genes showing the strongest conservation across teleost evolution are involved in brain functions and development, while further highlighting that SVs segregating in the studied farmed seabass population are likely to have substantial impacts on phenotypic variation, which could add value to the accuracy of genomic selection or ability to detect QTLs in ongoing aquaculture breeding efforts.

### A potential role for SVs in European seabass aquaculture domestication

Our discovery of SVs affecting conserved regions of European seabass genes with neurological and developmental functions motivated us to explore the role of SVs in aquaculture domestication. Bertolotti et al. (2020) [19] identified a strong enrichment for brain-expressed synaptic protein coding genes overlapped by SVs showing significantly different allele frequencies between farmed and wild Atlantic salmon, which were suggested to have promoted behavioural changes during domestication. Many of these SV alleles showed increases in frequency in farmed fish, consistent with a scenario where SVs deleterious in wild fish evolved under relaxed purifying or positive selection in farmed Atlantic salmon, owing to differences in rearing environment [19].

**Fig. 5:**
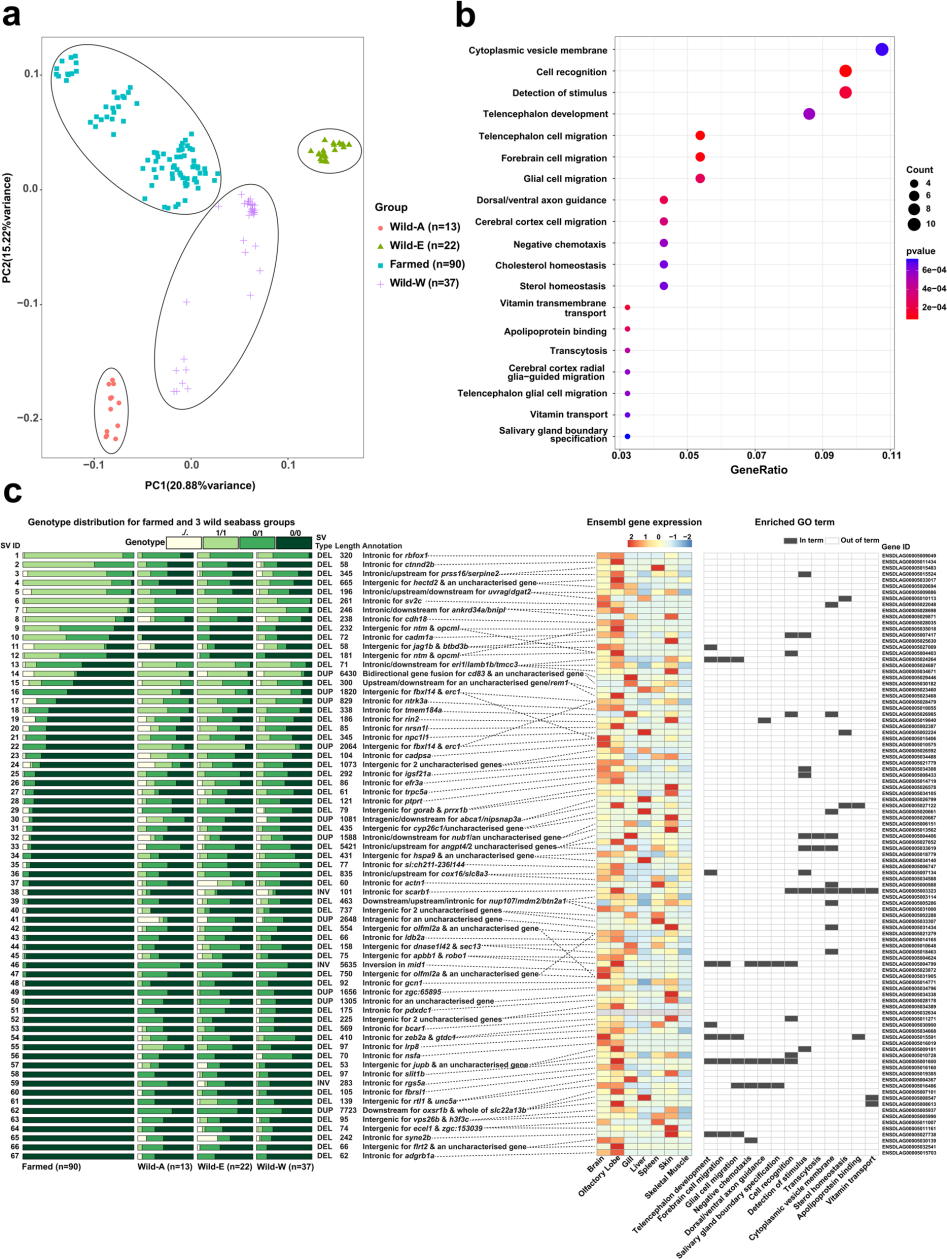
Comparative analysis of SVs in farmed and wild European seabass. These analyses were done using 41,336 SVs for *n* = 162 samples representing farmed and wild fish. The wild samples were from three geographical regions: Atlantic Ocean (Wild-A, *n* = 13), East Mediterranean (Wild-E, *n* = 22), West Mediterranean (Wild-W, *n* = 37). **a** PCA analysis of all SVs. **b** Enriched GO terms (*q* value/adjusted *p* value < 0.1) for 99 genes associated with 67 genetically differentiated SVs (top 5% F_ST_ values) common to the three comparisons: Farmed (*n* = 90) vs. Wild-A, Wild-E, or Wild-W. **c** Annotation of the 67 SVs and 99 associated genes. *Left*: relative SV genotype counts for each farmed vs. wild comparison along with annotations regarding SV type, length, genomic location and association to genes. *Right*: gene transcript per million (TPM) expression across a panel of tissues (standardized/scaled expression from −2 to + 2), and membership to enriched GO terms. Supporting results provided in Additional files Tables 14–18

To explore the role of SVs in European seabass domestication, we exploited published WGS data from three populations of wild European seabass [50, 51]. Using an identical pipeline to that applied with the farmed seabass SV dataset, we identified 38,408 high-confidence SVs in these samples (Additional file 14: Table 14). We will fully report this wild European seabass SV dataset in an independent study with distinct aim. Here, we focus on 41,336 SVs sharing identical locations in the reference genome derived from both datasets (see Methods) (Additional file 15: Table 15). For the current analysis, we compared genotypes for these merged SVs in samples comprising wild and farmed populations (Fig. 5).

Initially, we performed a principal component analysis (PCA) on the full set of SV genotypes to test the ability of SVs to capture expected genetic relationships across the sampled populations and individuals (Fig. 5a). This revealed genetically distinct groups of wild European seabass from different geographical regions, separated along PC1 and PC2, namely: Atlantic Ocean (‘wild-A’), West Mediterranean (‘wild-W’), and East Mediterranean (‘wild-E’), shown elsewhere to form the same genetically distinct groups using SNPs [52]. The farmed population forms a distinct group in the PCA, with sub-groups separated along PC1 and 2 (Fig. 5a), consistent with the mixed origins of the study population [33]. Overall, this analysis provides confidence in the quality of our SV discovery and genotyping pipeline, with respect to its ability to recover known population structure observed elsewhere using SNPs.

We next calculated the fixation index (F_ST_) using genotypes for the 41,336 SVs in three comparisons: i) farmed (*n* = 90) vs. Wild-A (*n* = 13), ii) farmed (*n* = 90) vs. Wild-E (*n* = 22) and iii) farmed (*n* = 90) vs. Wild-W (*n* = 37). We retained only SVs identified within the top 5% F_ST_ values in all three comparisons, which were filtered to retain 67 SVs showing the same direction of allele frequency change in each case (Additional file 16: Table 16). This strategy reduces the confounding effects of population structure between the wild geographical groups, which would have occurred in a global analysis of all farmed vs. all wild fish.

SnpEff annotation revealed that the 67 SVs were associated with 99 unique genes (Additional file 16: Table 16). We performed a GO analysis using these genes (Fig. 5b; Additional file 17: Table 17), mapped differences in genotype frequency for the associated SVs in the three farmed vs. wild comparisons, quantified each gene's tissue-specific expression (Fig. 5c) and summarised the known roles of orthologs from work in other species (Additional file 18: Table 18).

GO analysis revealed enrichment of the terms “*Telencephalon cell migration*”, “*Telencephalon development*”, “*Cerebral cortex cell migration*” and “*Telencephalon glial cell migration*” (Fig. 5b; Additional file 17: Table 17). The telencephalon (i.e., cerebrum/forebrain, which includes the cerebral cortex) is centrally involved in behaviour, neural plasticity and cognition in fish [53] and a primary target for domestication in diverse farmed animal species [54, 55]. Telencephalon-associated enriched GO terms were explained by an overlapping set of eight genes, including *slit1b*, encoding slit Homolog 1, involved in axon guidance and neural development, playing a key role in directing neuron migration and connectivity [56], as well as stress and behavioural responses [57]. A 97 bp intronic deletion in *slit1b* was present in moderate allele frequencies in each wild population (0.15, 0.19, 0.13 for Wild-A, Wild-E, Wild-W, respectively), but absent in the farmed population. *robo1* was another gene explaining enrichment of telencephalon-associated GO terms (Fig. 5c), encoding roundabout guidance receptor 1, which mediates nerve axon guidance through its interaction with Slit proteins, and is crucial for neural circuit formation and development [58]. However, it should be noted that the 75 bp deletion associated with *robo1* was ~ 77 kb upstream (Additional file 16: Table 16). Similar to *slit1b*, this SV was almost absent in the farmed population, but present in moderate frequencies in the wild groups (0.25, 0.30, 0.15 for Wild-A, Wild-E, Wild-W, respectively). It is notable that vertebrate Slit proteins form complexes with Robo proteins to dictate neuron axon guidance in the olfactory bulb of the telencephalon [59], as *slit1b* and *robo1* were specifically expressed in the seabass olfactory lobe (Fig. 5c). Another gene explaining the telencephalon-associated enriched GO terms was *lrp8*, encoding low-density Lipoprotein receptor-related protein 8, which was also highly expressed in the seabass brain and olfactory lobe (Fig. 5c). LRP8 is the receptor for Reelin, an interaction that activates signalling pathways essential to synaptic plasticity, learning and memory formation [60]. A 97 bp intronic deletion within *lrp8* was nearly absent in farmed fish, but present at moderate to high frequencies in the wild groups (0.63, 0.41, 0.15 for Wild-A, Wild-E, Wild-W, respectively). Interestingly, an intronic deletion in *lrp8* showed significantly different allele frequencies between farmed and wild Atlantic salmon [19], hinting at convergent evolution.

Beyond genes explaining enrichment of the telencephalon-associated GO terms, many others within the 99 domestication-associated genes have roles associated with the brain and nervous system, including synapses and behaviour (Fig. 5c; Additional file 18: Table 18). For example, *cadm1a*, contributing to enriched term *“detection of stimulus”*, was expressed highly in the brain and olfactory lobe (Fig. 5c), consistent with its role as a synapse adhesion molecule [61]. A 78 bp intronic deletion in *cadm1* showed an increase in allele frequency in the farmed seabass population (0.58) compared to the wild groups (0.08, 0.11 and 0.25 for Wild-A, Wild-E, Wild-W, respectively). While not contributing to any enriched terms, a 320 intronic deletion in *rbfox1*, encoding RNA splicing factor RNA Binding Fox-1 Homolog 1, was highly differentiated between farmed and wild populations, being almost fixed in the former (allele frequency: 0.95) (Fig. 5c). Again, *rbfox1* was specifically expressed in the brain and olfactory lobe (Fig. 5c), consistent with the human ortholog’s known role as a neuron-specific factor linked to a range of neurological conditions, including autism [62]. Further genes associated with SVs showing genetic differentiation between wild and farmed seabass and expressed specifically in brain and/or olfactory lobe included: *ctnnd2* (harboring a 58 bp intronic deletion), encoding adhesive junction associated synaptic protein—linked to neurodevelopmental disorders in humans, which regulates forebrain neuronal organisation and behavioural phenotypes in zebrafish [63]; *sv2* (harboring a 261 bp intronic deletion) encoding a synaptic vesicle protein; *btbd3b*, which in mammals controls the orientation of neuron dendrites in the cerebral cortex [64]; *nrsn1l*, a neuron-specific gene encoding a protein supporting axon and dendrite extension [65]; and *nsf*, encoding a protein with multifaceted synaptic and neuronal functions [66]; among other examples (Fig. 5c; Additional file 18: Table 18).

Beyond the signal of brain-associated functions among the 99 genes, a small number of additional enriched terms are interesting in the context of domestication. Contributing to enriched term ‘*cholesterol homeostasis’*, we identified a 345 bp intronic deletion in *npc1l1*, present in much higher frequencies in the wild groups (Fig. 5c). In humans, this gene plays a key role in regulating hepatic cholesterol [67], consistent with its liver-specific expression in seabass (Fig. 5c). Several other genes impacted by the 67 SVs had roles in lipid storage, transport or metabolism, including *abca1* and *dgat2* (Additional file 18: Table 18).

Two of the 67 SVs were annotated as high-impact variants by SnpEff, while only one overlapped an evolutionary conserved GERP region (Additional file 16: Table 16). One of the high-impact SVs was a 6,430 bp duplication that overlapped two genes, spanning a non-coding exon and intronic region (containing a predicted enhancer) of *cd83*, as well as the last coding exon of a gene that is uncharacterized in Ensembl (ENSDLAG00005030182), but named as tripartite motif-containing protein 16-like by NCBI. This duplication is predicted to cause a bidirectional gene fusion. Both genes are most highly expressed in the seabass gill and the duplication is present at a much lower frequency in the farmed population (Fig. 5c). In other teleost species, *cd83* has an immunological function in antigen-presenting dendritic cells in gills, and is strongly upregulated during vaccination and pathogen challenge [68, 69]. It will be interesting to test whether the identified duplication impacts immunological functions in European seabass carrying different genotypes.

Finally, we asked if any of the genetically differentiated SVs between wild and farmed seabass overlapped with Ensembl predicted regulatory elements. Among the 67 SVs, 6 overlapped with unique predicted regulatory elements, specifically 3 enhancers and 3 open chromatin regions. One open chromatin region (ENSDLAR00000234746) that shows predicted activity specifically during gastrulation, was disrupted by a 66 bp deletion located on CAJNNU010000003.1, present exclusively in wild seabass populations (allele frequency: 0.27, 0.19 and 0.10 for Wild-A, Wild-E, Wild-W, respectively). This region sits 90 Kb upstream of *flrt2* (ENSDLAG00005030139), a conserved gene with key functions during early embryonic development, including heart morphogenesis [70]. Another notable SV was a 410 bp deletion on CAJNNU010000006.1, which disrupted an intronic enhancer (ENSDLAR00000182270) embedded in two overlapping genes, *zeb2a* (ENSDLAG00005030900) and *gtdc1* (ENSDLAG00005034668), both with known roles in brain development and neurological functions [71, 72]. This SV was common in wild seabass (allele frequency: 0.33, 0.34 and 0.40 for Wild-A, Wild-E, Wild-W, respectively), but rare in the farmed population (allele frequency: 0.001). The remaining SVs were duplications, with one (6,430 bp deletion) discussed in the last paragraph (impacting *cd83*). We further identified a 1,656 bp duplication on CAJNNU010000016 that fully overlapped an open chromatin region (ENSDLAR00000252590) showing activity across embryonic development, found in the first exon of a poorly-characterised gene annotated as zgc:65,895 (ENSDLAG00005034796). This SV was again rare in the farmed population (allele frequency: 0.01) but common in each wild population (allele frequency: 0.27, 0.23 and 0.18 for Wild-A, Wild-E, Wild-W, respectively). Finally, a 1,588 bp duplication on scaffold CAJNNU010000021.1 fully overlapped with enhancer ENSDLAR00000140351, which shows predicted activity in gill, gonad, and liver. This enhancer is located in the first intron of *nub1* (ENSDLAG00005006151), a gene encoding NEDD8 ultimate buster 1, an interferon stimulated molecule that downregulates *NEDD8* [73], a gene encoding a ubiquitin-protein considered responsible for variation in resistance to infectious pancreatic necrosis in farmed Atlantic salmon [74]. Again, this duplication was rare in the farmed population (allele frequency: 0.02), but common in the wild populations (allele frequency: 0.21, 0.32 and 0.23 for Wild-A, Wild-E, Wild-W, respectively).

Collectively, these findings highlight potential functional impacts of SVs on key regulatory elements and associated genes, underscoring their relevance in understanding phenotypic divergence between wild and farmed populations.

## Conclusions

SVs represent a fundamental component of genomic diversity in all species, and play a major role in shaping the genetic architecture of diverse traits. This study provides a comprehensive atlas of high-confidence SVs in the European seabass genome, significantly advancing understanding of genomic and population genetic diversity in an economically important teleost. The SV datasets reported here can be uptaken in future genomic studies of European seabass, including as a high-quality reference panel that can be imputed to larger populations [75], or taken forward as candidate causal variants in known (or to be defined) QTL regions associated with traits of interest in aquaculture practise, contributing to future efforts to enhance growth rate, disease resistance and stress tolerance. However, we acknowledge that the dataset presented is unlikely representative of the true diversity of SVs present in this species, while our results provide only a first glimpse into the potential impacts of these variants. In this respect, we also discovered a range of candidate genes and regulatory elements potentially associated with European seabass domestication that warrant further investigation, pointing to a role for SVs in targeting brain-expressed genes influencing behaviour and neurological disorders, mirroring the proposed genomic basis for behavioural domestication in other farmed teleosts [19]. Past work in European seabass implied epigenetic processes as a key factor in the early stages of domestication, targeting the developing neural crest [76]. In the future, integrated analyses of genetic and epigenetic variation comparing farmed and wild European seabass populations would help disentangle how these key processes have co-shaped domestication across different generations of aquaculture. Further work will also be required to understand the extent to which SVs disrupting genes with functions in development and the nervous system are shaping variation in commercial or welfare traits within farmed European seabass populations, and whether this variation can be positively harnessed in selective breeding.

While our analysis leverages robust methods, we acknowledge limitations inherent to short-read WGS, which fails to accurately resolve complex and larger SVs and significantly underestimates SV diversity according to studies in other fish species [20]. Moreover, our SV-plaudit curation pipeline, while reducing false positives [19], may remove real SVs lacking strong visual evidence. Future research employing long-read sequencing technologies are needed to expand and improve SV detection, offering deeper insights into the complex spectrum of genomic diversity in European seabass.

Overall, this work provides a high-quality SV atlas for European seabass, providing important new insights into the functional implications of SVs in this species, including in relation to aquaculture domestication. This marks a significant advancement in European seabass genomics and a key reference for research aimed at improving breeding programmes, enhancing aquaculture productivity, and understanding evolutionary and ecological dynamics in wild populations.

## Methods

### Samples for whole genome sequencing in farmed European seabass

The WGS samples used to detect SVs in farmed European seabass were sourced from the AQUA-FAANG project (fully described in Mukiibi et al. 2025) [33]. These samples comprised *n* = 90 individuals from an Italian breeding population, representing 50 parent animals (25 males and 25 females crossed in a fully factorial design), and 40 offspring resulting from crossing of the parents, and were selected to capture the genetic structure of the parental stock. Briefly, total genomic DNA was extracted from fin clip tissue of each sample and subsequently processed to generate sequencing libraries. The libraries were then paired-end sequenced on Illumina’s NovaSeq 6000 platform (Illumina, California, San Diego, USA) with read length of 150 bp [77].

### Mapping and quality assessment

The WGS data was assessed for sequencing quality using FastQC v0.11.9 (https://github.com/s-andrews/FastQC). Trimmomatic v0.38 [78] was used to trim sequencing adaptors, low quality sequencing reads, and remove reads < 50 bp. The retained clean reads were mapped to the unmasked annotated European seabass reference genome (dlabrax2021/GCA_905237075.1) downloaded from the Ensembl Genome Browser (Release 112) (https://www.ensembl.org/Dicentrarchus_labrax/Info/Index), using the Burrows-Wheeler Aligner (BWA) v0.7.17 [79]. Samtools v.1.13 [80] was used to convert alignment files to BAM format, and Mosdepth v0.3.3 [77] to estimate genome-wide WGS depth for each sample (average = 18.5x; range = 13.2–28.7x).

### SV detection and genotyping

We used an established pipeline for SV detection and genotyping based on Lumpy v0.2.7 [81] within the Smoove framework (https://github.com/brentp/smoove), based on Bertolotti et al. (2020) and Liu et al. (2021) [19, 21]. Lumpy exploits signals derived from aligned WGS read depth, split reads and discordant paired-end reads to predict breakpoints and detect deletions, duplications, inversions and translocations. Within Smoove v0.2.5, SVtyper v0.7.0 [82] was used to genotype SVs, creating a VCF file containing all raw genotyped SVs.

To reduce false positive calls, a series of bioinformatic filtering steps were applied to the raw SV calls. Specifically, we: i) removed any SVs detected in unplaced scaffolds to retain SVs located on the largest chromosomal scaffolds (CAJNNU010000001.1 to CAJNNU010000024.1), which are well annotated for downstream analysis and interpretations; ii) removed translocations, which are challenging to validate by manual curation, as done by other authors [19, 21]; iii) removed SVs overlapping gaps in scaffolds or located in ‘high-depth’ regions (defined here as showing > 100 × sequencing depth), which were shown elsewhere to be dominated by false-positive SV calls [19], iv) used Duphold v0.2.1 to filter low-quality SVs, involving the assessment of sequencing depth around SV breakpoints, an approach that has been shown to increase detection sensitivity by strongly biasing the retention of true SVs [83]. During this step, SV genotypes were replaced as './.' (i.e., missing data) in the VCF file in cases where fold-change for the variant depth relative to flanking regions (DHFFC) parameter was > 0.7 for deletions, < 1.3 for duplications, and between 0.7 and 1.3 for inversions; v) removing any SVs with allele count <  = 2 and/or genotype call rate < 80%, as done by other authors [21].

### SV validation

SVs that passed the above filters were processed through a final visual curation step using SV-plaudit [84]. SV-plaudit is a framework for generating images to visually validate SVs via Samplot v1.3.0 [85] and has been demonstrated to substantially reduce false positives, and preserve true SVs [19, 85]. Curated images were uploaded to a cloud-based repository and a dedicated website was deployed for viewing and scoring every SV, with resultant scores extracted for analysis and filtering. The input for SV-plaudit was the reference genome, alignment BAM files, and VCF file containing SVs. SV images were uploaded to Amazon Web Services using a Python script (https://github.com/jbelyeu/PlotCritic/blob/f1b4119511067787070245eacf2c9ec8c5f4cb1e/upload.py). For each SV, this included a random selection of seven samples, including, when available, two homozygote samples lacking the SV (i.e., 0/0 genotype), two heterozygote samples with the SV (i.e., 0/1 genotype), and three homozygote samples with the SV (i.e., 1/1 genotype).

### SV annotation approach

For each high-confidence SV retained after SV-plaudit validation, VCFtools v0.1.16 [86] was used to estimate allele frequencies. SnpEff v5.1d [87] was used to predict the impact of SVs on annotated genomic features in the European seabass genome, including genes in the Ensembl genebuild annotation [88]. RepeatMasker v4.1.5 [89] was used to annotate repeat elements including transposable elements (TEs) with the “-*species dicentrarchus labrax*” option. The intersect function of Bedtools v2.30.0 [90] was used to examine the relationship between repeat elements and SVs using a minimum 50% reciprocal overlap criteria with the parameter “*-wo -f 0.50 -r*”.

### Orthology prediction

Through Ensembl Release 112, BioMart (https://www.ensembl.org/biomart/martview) was used to extract information to support annotation of genes associated with SVs, including features derived from the Ensembl Compara resource [91]. We downloaded predicted orthologues of European seabass genes from three spined stickleback *Gasterosteus aculeatus* (GAculeatus_UGA_version5; GCA_016920845.1), Atlantic salmon *Salmo salar* (Ssal_v3.1; GCA_905237065.2), zebrafish *Danio rerio* (GRCz11; GCA_000002035.4), human *Homo sapiens* (GRCh38.p14; GCA_000001405.29) and mouse *Mus musculus* (GRCm39; GCA_000001635.9) to provide candidate name/symbol descriptions and support interpretations for European seabass genes lacking symbols/names.

### Overlapping SVs with evolutionarily conserved genomic regions

Using BioMart, we downloaded evolutionary constrained elements in the European seabass genome, which were predicted using Genomic Evolutionary Rate Profiling (GERP) [37] using an Enredo-Pecan-Ortheus (EPO) based whole genome alignment (described in Herrero et al. 2016) [91] including 65 actinopterygian fish, produced by Ensembl (obtained from FTP site: https://ftp.ensembl.org/pub/release-112/bed/ensembl-compara/65_fish.gerp_constrained_element/gerp_constrained_elements.dicentrarchus_labrax.bb). The location of these evolutionarily constrained elements was overlapped with SV locations using Bedtools.

### Protein domain analyses for genes affected by high impact SVs

We downloaded predicted Interpro domains [92] for all European seabass genes from Ensembl BioMart, which were used to identify protein domains overrepresented among genes affected by SVs annotated as high-impact by SnpEff. Specifically, separately for every Interpro domain, we compared the proportion of genes affected by high impact SVs among all genes associated with high impact SVs, versus the proportion of genes not affected by high impact SVs among all genes not associated with high impact SVs, using a Fisher’s exact test, followed by multiple testing correction using the Benjamini–Hochberg procedure to obtain adjusted *p* values (Additional File 9, Table 9).

### Comparative analysis of SVs in farmed and wild European seabass

To investigate the role of SVs in European seabass domestication, we compared the dataset produced for the farmed individuals described above (i.e., 21,248 high-confidence SV dataset, *n* = 90 WGS samples) with a new dataset produced independently for a separate group of wild seabass. In brief, this dataset comprised 38,408 SVs detected from paired-end WGS data for *n* = 80 wild European seabass from diverse wild populations (*n* = 13 from Atlantic Ocean, *n* = 22 from East Mediterranean, *n* = 37 from West Mediterranean and *n* = 8 from known F1 hybrids; see below) using an SV detection, filtering and genotyping strategy identical to the section ‘SV detection and genotyping’. These WGS samples represent three phylogeographic lineages of European seabass and were downloaded from NCBI accessions: PRJNA628166 and PRJNA472842. PRJNA628166 provided *n* = 58 samples described in Duranton et al. (2020) [50] while PRJNA472842 provided *n* = 22 samples described by Duranton et al. (2019) [51]. Following the approach described above for farmed population WGS samples, we calculated average WGS depth of 31.3x (range = 10.54–96.03x) across all wild samples derived from both datasets using Mosdepth. WGS depth was also calculated separately for PRJNA628166 (*n* = 58; average = 37.6x; range = 17.6–96.0x) and PRJNA472842 (*n* = 22; average = 14.5x, range = 10.5–20.3x).

For the purpose of comparative analysis, we repeated the first steps of the Smoove SV calling and genotyping pipeline across all *n* = 170 WGS samples (i.e., *n* = 90 farmed, *n* = 80 wild). The raw SVs were filtered using steps ‘i’ and ‘ii’ described above in the section ‘SV detection and genotyping’. Next, an integrated set of high-confidence genotyped SVs was retrieved using Bedtools by overlapping the locations of SVs from the high-confidence SV datasets (i.e., 21,428 and 38,408 SVs independently generated for the farmed and wild groups, respectively) and the integrated SV calls from the *n* = 170 sample dataset using a 100% reciprocal overlap criteria. This led to a final integrated/merged dataset of 41,336 SVs capturing 94.5% and 95.2% of the original high-confidence SVs from the farmed and wild SV datasets, respectively.

Principal component analysis (PCA) was performed to compare the genetic relationships of both farmed and wild WGS samples using PLINK v1.90b6.21 [93], which confirmed three expected phylogeographic lineages in the wild seabass samples: Atlantic Ocean (Wild-A), East Mediterranean (Wild-E) and West Mediterranean (Wild-W). Prior to this analysis, and in subsequent comparisons, we removed *n* = 8 experimentally-produced F1 hybrids between the different geographical groups [50].

Next, employing the integrated 41,336 SV genotypes, VCFtools v0.1.16 was used to calculate the fixation index (F_ST_) in three pairwise combinations: i) farmed (*n* = 90) vs. Wild-A (*n* = 13), ii) farmed (*n* = 90) vs. Wild-E (*n* = 22) and iii) farmed (*n* = 90) vs. Wild-W (*n* = 37). With the goal of reducing the effect of population genetic structure among the wild populations, we took forward 67 SVs as candidates associated with domestication on the basis of: i) being in the top 5% of F_ST_ values in all three comparisons, and ii) having the same directionality of allele frequency change in all three comparisons of farmed and wild individuals. Using SnpEff annotations, 99 genes associated with the 67 candidate SVs were identified before their expression was explored using RNA-seq data provided by Ensembl (https://ftp.ensembl.org/pub/release-112/bamcov/dicentrarchus_labrax/genebuild/) for brain, olfactory lobe, gill, liver, spleen, skin and skeletal muscle. FeatureCounts v2.0.8 [94] was then used to generate transcripts per million (TPM) information. Additionally, Gene Ontology enrichment tests were performed on the 98 candidate genes, as described in the next section.

### Gene set enrichment tests

Gene Ontology (GO) terms and Kyoto Encyclopedia of Genes and Genomes (KEGG) pathways were annotated for all European seabass coding genes (n = 24,969) using eggNOG-Mapper [95]. 22,648 genes were annotated with GO terms and 10,747 genes were annotated with KEGG pathway information. GO term and KEGG pathway enrichment tests were then performed using the clusterProfiler R package [96] on the following datasets: i) high-impact farmed seabass SVs annotated by SnpEff (among the 21,428 high-confidence SVs), ii) all SVs within the 21,428 high-confidence farmed seabass SVs that overlapped with GERP (evolutionarily conserved) regions described above, iii) as for ‘ii’, but restricted to GERP regions < 10 kb in length, iv) genes associated with SVs representing candidates associated with domestication (last section). For tests i-iii, the background was set as all genes associated by SNPeff with the 21,428 high-confidence SVs from the farmed seabass group. For tests iv, the background was set as all genes associated by SNPeff with the 41,336 high-confidence SVs from the integrated wild and farmed seabass group. We accepted GO terms as significantly enriched at adjusted *p* and *q v*alues < 0.05 for tests i-iii and at < 0.1 for test iv, owing to the small number of genes involved.

### Plots and statistics

All plots used were based on ggplot2 [97], except for Fig. 1b, which was made using CMplot [98], Fig. 2b, which was made using UpSetR [99], and part of Fig. 5c, which was made using pheatmap [100]. All reported statistical tests were performed in Python3 using the "stats" module. The Ensembl Genome Browser was used to visualise specific SVs in relation to annotated features in the European seabass genome. Images from Ensembl were exported as PDFs, with minor edits made in GIMP v2.10.8.

## Supplementary Information

Additional file 1.

Additional file 2.

Additional file 3.

Additional file 4.

Additional file 5.

Additional file 6.

Additional file 7.

Additional file 8.

Additional file 9.

Additional file 10.

Additional file 11.

Additional file 12.

Additional file 13.

Additional file 14.

Additional file 15.

Additional file 16.

Additional file 17.

Additional file 18.

## Data Availability

The whole genome sequencing datasets used in this study are available through NCBI with the following BioProject accession numbers: PRJNA1110973 [33], PRJNA628166 [50] and PRJNA472842 [51]. All code used in this study is available on GitHub (https://github.com/JiaoZexin/European-Seabass-SV-landscape).

## Abbreviations

SV: Structural variant
SNP: Single nucleotide polymorphism
QTL: Quantitative trait loci
WGS: Whole genome sequencing
MAF: Minor allele frequency
UTR: Untranslated region
TE: Transposable element
LINEs: Long interspersed nuclear elements
SINEs: Short interspersed nuclear elements
GERP: Genomic Evolutionary Rate Profiling
PCA: Principal component analysis
Wild-A: Wild seabass samples from Atlantic Ocean
Wild-E: Wild seabass samples from East Mediterranean
Wild-W: Wild seabass samples from West Mediterranean
GO: Gene Ontology
KEGG: Kyoto Encyclopedia of Genes and Genomes
F_ST_: Fixation index
TPM: Transcripts per million
DHFFC: Fold-change for the variant depth relative to Flanking regions
EPO: Enredo-Pecan-Ortheus

## References

[1] Alkan C, Coe BP, Eichler EE. Genome structural variation discovery and genotyping. Nat Rev Genet. 2011;12:363–76. 10.1038/nrg2958. 21358748 10.1038/nrg2958 PMC4108431

[2] Kosugi S, Momozawa Y, Liu X, Terao C, Kubo M, Kamatani Y. Comprehensive evaluation of structural variation detection algorithms for whole genome sequencing. Genome Biol. 2019;20:8–11. 10.1186/s13059-019-1720-5. 31159850 10.1186/s13059-019-1720-5 PMC6547561

[3] Chiang C, Scott AJ, Davis JR, Tsang EK, Li X, Kim Y, et al. The impact of structural variation on human gene expression. Nat Genet. 2017;49:692–9. 10.1038/ng.3834. 28369037 10.1038/ng.3834 PMC5406250

[4] Ho SS, Urban AE, Mills RE. Structural variation in the sequencing era. Nat Rev Genet. 2020;21:171–89. 10.1038/s41576-019-0180-9. 31729472 10.1038/s41576-019-0180-9 PMC7402362

[5] Weischenfeldt J, Symmons O, Spitz F, Korbel JO. Phenotypic impact of genomic structural variation: insights from and for human disease. Nat Rev Genet. 2013;14:125–38. 10.1038/nrg3373. 23329113 10.1038/nrg3373

[6] Li Y, Roberts ND, Wala JA, Shapira O, Schumacher SE, Kumar K, et al. Author Correction: Patterns of somatic structural variation in human cancer genomes. Nature. 2023;578:112–21. 10.1038/s41586-022-05597-x. 10.1038/s41586-022-05597-x PMC9931568 36697835

[7] Alonge M, Wang X, Benoit M, Soyk S, Pereira L, Zhang L, et al. Major impacts of widespread structural variation on gene expression and crop improvement in tomato. Cell. 2020;182:145-161.e23. 10.1016/j.cell.2020.05.021. 32553272 10.1016/j.cell.2020.05.021 PMC7354227

[8] Scott AJ, Chiang C, Hall IM. Structural variants are a major source of gene expression differences in humans and often affect multiple nearby genes. Genome Res. 2021;31:2249–57. 10.1101/gr.275488.121. 34544830 10.1101/gr.275488.121 PMC8647827

[9] Lu Y, Tian Y, Shen R, Yao Q, Wang M, Chen M, et al. Targeted, efficient sequence insertion and replacement in rice. Nat Biotechnol. 2020;38:1402–7. 10.1038/s41587-020-0581-5. 32632302 10.1038/s41587-020-0581-5

[10] Porubsky D, Sanders AD, Höps W, Hsieh P, Sulovari A, Li R, et al. Recurrent inversion toggling and great ape genome evolution. Nat Genet. 2020;52:849–58. 10.1038/s41588-020-0646-x. 32541924 10.1038/s41588-020-0646-x PMC7415573

[11] Bothmer A, Gareau KW, Abdulkerim HS, Buquicchio F, Cohen L, Viswanathan R, et al. Detection and modulation of DNA translocations during multi-gene genome editing in T cells. CRISPR J. 2020;3:177–87. 10.1089/crispr.2019.0074. 32584143 10.1089/crispr.2019.0074

[12] Fueyo R, Judd J, Feschotte C, Wysocka J. Roles of transposable elements in the regulation of mammalian transcription. Nat Rev Mol Cell Biol. 2022;23:481–97. 10.1038/s41580-022-00457-y. 35228718 10.1038/s41580-022-00457-y PMC10470143

[13] Choi J, Chen W, Suiter CC, Lee C, Chardon FM, Yang W, et al. Precise genomic deletions using paired prime editing. Nat Biotechnol. 2022;40:218–26. 10.1038/s41587-021-01025-z. 34650269 10.1038/s41587-021-01025-z PMC8847327

[14] Birchler JA, Yang H. The multiple fates of gene duplications: deletion, hypofunctionalization, subfunctionalization, neofunctionalization, dosage balance constraints, and neutral variation. Plant Cell. 2022;34:2466–74. 10.1093/plcell/koac076. 35253876 10.1093/plcell/koac076 PMC9252495

[15] Kuzmin E, Taylor JS, Boone C. Retention of duplicated genes in evolution. Trends Genet. 2022;38:59–72. 10.1016/j.tig.2021.06.016. 34294428 10.1016/j.tig.2021.06.016 PMC8678172

[16] Almarri MA, Bergström A, Prado-Martinez J, Yang F, Fu B, Dunham AS, et al. Population structure, stratification, and introgression of human structural variation. Cell. 2020;182:189–99. 10.1016/j.cell.2020.05.024. 32531199 10.1016/j.cell.2020.05.024 PMC7369638

[17] Li N, He Q, Wang J, Wang B, Zhao J, Huang S, et al. Super-pangenome analyses highlight genomic diversity and structural variation across wild and cultivated tomato species. Nat Genet. 2023;55:852–60. 10.1038/s41588-023-01340-y. 37024581 10.1038/s41588-023-01340-y PMC10181942

[18] Collins RL, Brand H, Karczewski KJ, Zhao X, Alföldi J, Francioli LC, et al. A structural variation reference for medical and population genetics. Nature. 2020;581:444–51. 10.1038/s41586-020-2287-8. 32461652 10.1038/s41586-020-2287-8 PMC7334194

[19] Bertolotti AC, Layer RM, Gundappa MK, Gallagher MD, Pehlivanoglu E, Nome T, et al. The structural variation landscape in 492 Atlantic salmon genomes. Nat Commun. 2020. 10.1038/s41467-020-18972-x. 33056985 10.1038/s41467-020-18972-x PMC7560756

[20] Lecomte L, Árnyasi M, Ferchaud A, Kent M, Lien S, Stenløkk K, et al. Investigating structural variant, indel and single nucleotide polymorphism differentiation between locally adapted Atlantic salmon populations. Evol Appl. 2024;17:e13653. 10.1111/eva.13653. 38495945 10.1111/eva.13653 PMC10940791

[21] Liu S, Gao G, Layer RM, Thorgaard GH, Wiens GD, Leeds TD, et al. Identification of High-Confidence Structural Variants in Domesticated Rainbow Trout Using Whole-Genome Sequencing. Front Genet. 2021;12 February:1–9. 10.3389/fgene.2021.639355. 10.3389/fgene.2021.639355 PMC7959816 33732289

[22] Mérot C, Stenløkk KSR, Venney C, Laporte M, Moser M, Normandeau E, et al. Genome assembly, structural variants, and genetic differentiation between lake whitefish young species pairs (*Coregonus* sp.) with long and short reads. Mol Ecol. 2023;32:1458–77. 10.1111/mec.16468. 35416336 10.1111/mec.16468

[23] Ruigrok M, Xue B, Catanach A, Zhang M, Jesson L, Davy M, et al. The relative power of structural genomic variation versus SNPs in explaining the quantitative trait growth in the marine teleost *Chrysophrys auratus*. Genes. 2022;13:1129. 10.3390/genes13071129. 35885912 10.3390/genes13071129 PMC9320665

[24] Jiao Z, Tian Y, Hu B, Li Q, Liu S. Genome structural variation landscape and its selection signatures in the fast-growing strains of the Pacific oyster, *Crassostrea gigas*. Mar Biotechnol. 2021;23:736–48. 10.1007/s10126-021-10060-5. 10.1007/s10126-021-10060-5 34498173

[25] Vandeputte M, Gagnaire P, Allal F. The European sea bass: a key marine fish model in the wild and in aquaculture. Anim Genet. 2019;50:195–206. 10.1111/age.12779. 30883830 10.1111/age.12779 PMC6593706

[26] FAO. The State of World Fisheries and Aquaculture 2022. FAO; 2022. 10.4060/cc0461en.

[27] Palaiokostas C, Cariou S, Bestin A, Bruant J-S, Haffray P, Morin T, et al. Genome-wide association and genomic prediction of resistance to viral nervous necrosis in European sea bass (*Dicentrarchus labrax*) using RAD sequencing. Genet Sel Evol. 2018;50:30. 10.1186/s12711-018-0401-2. 29884113 10.1186/s12711-018-0401-2 PMC5994081

[28] Peñaloza C, Manousaki T, Franch R, Tsakogiannis A, Sonesson AK, Aslam ML, et al. Development and testing of a combined species SNP array for the European seabass (*Dicentrarchus labrax*) and gilthead seabream (*Sparus aurata*). Genomics. 2021;113:2096–107. 10.1016/j.ygeno.2021.04.038. 33933591 10.1016/j.ygeno.2021.04.038 PMC8276775

[29] Vela-Avitúa S, Thorland I, Bakopoulos V, Papanna K, Dimitroglou A, Kottaras E, et al. Genetic basis for resistance against viral nervous necrosis: GWAS and potential of genomic prediction explored in farmed European sea bass (*Dicentrarchus labrax*). Front Genet. 2022. 10.3389/fgene.2022.804584. 35401661 10.3389/fgene.2022.804584 PMC8992836

[30] Oikonomou S, Kazlari Z, Papapetrou M, Papanna K, Papaharisis L, Manousaki T, et al. Genome wide association (GWAS) analysis and genomic heritability for parasite resistance and growth in European seabass. Aquac Rep. 2022;24:101178. 10.1016/j.aqrep.2022.101178.

[31] Oikonomou S, Samaras A, Tekeoglou M, Loukovitis D, Dimitroglou A, Kottaras L, et al. Genomic selection and genome-wide association analysis for stress response, disease resistance and body weight in European seabass. Animals. 2022;12:277. 10.3390/ani12030277. 35158601 10.3390/ani12030277 PMC8833606

[32] Griot R, Allal F, Phocas F, Brard-Fudulea S, Morvezen R, Bestin A, et al. Genome-wide association studies for resistance to viral nervous necrosis in three populations of European sea bass (*Dicentrarchus labrax*) using a novel 57k SNP array DlabChip. Aquaculture. 2021;530:735930. 10.1016/j.aquaculture.2020.735930.

[33] Mukiibi R, Ferraresso S, Franch R, Peruzza L, Dalla Rovere G, Babbucci M, et al. Integrated functional genomic analysis identifies regulatory variants underlying a major QTL for disease resistance in European sea bass. BMC Biol. 2025;23:75. 10.1186/s12915-025-02180-4. 40069747 10.1186/s12915-025-02180-4 PMC11899128

[34] Beck CR, Garcia-Perez JL, Badge RM, Moran JV. LINE-1 elements in structural variation and disease. Annu Rev Genomics Hum Genet. 2011;12(1):187–215. 10.1146/annurev-genom-082509-141802. 21801021 10.1146/annurev-genom-082509-141802 PMC4124830

[35] D’Souza UM, Craig IW. CHAPTER 1.2 - Genetic Organization of the Serotonergic System. In: Müller CP, Jacobs BLBT-H of BN, editors. Handbook of the Behavioral Neurobiology of Serotonin. Elsevier; 2010. p. 23–50. 10.1016/S1569-7339(10)70070-9.

[36] Vandooren J, Itoh Y. Alpha-2-macroglobulin in inflammation, immunity and infections. Front Immunol. 2021;12:803244. 10.3389/fimmu.2021.803244. 34970276 10.3389/fimmu.2021.803244 PMC8712716

[37] Cooper GM, Stone EA, Asimenos G, Green ED, Batzoglou S, Sidow A. Distribution and intensity of constraint in mammalian genomic sequence. Genome Res. 2005;15:901–13. 10.1101/gr.3577405. 15965027 10.1101/gr.3577405 PMC1172034

[38] Moser HW, Mahmood A, Raymond GV. X-linked adrenoleukodystrophy. Nat Clin Pract Neurol. 2007;3:140–51. 10.1038/ncpneuro0421. 17342190 10.1038/ncpneuro0421

[39] Limoni G, Niquille M. Semaphorins and plexins in central nervous system patterning: the key to it all? Curr Opin Neurobiol. 2021;66:224–32. 33513538 10.1016/j.conb.2020.12.014

[40] Matsumoto T, Miyake N, Watanabe Y, Yamanaka G, Oana S, Ogiwara M, et al. X-linked adrenoleukodystrophy with partial deletion of ALD due to fusion with the neighbor gene, PLXNB3. Am J Med Genet Part A. 2005;138:300–2. 10.1002/ajmg.a.30951. 10.1002/ajmg.a.30951 16152637

[41] Shankar P, Dasgupta S, Hahn ME, Tanguay RL. A review of the functional roles of the zebrafish aryl hydrocarbon receptors. Toxicol Sci. 2020;178:215–38. 10.1093/toxsci/kfaa143. 32976604 10.1093/toxsci/kfaa143 PMC7706399

[42] Michetti F, D’Ambrosi N, Toesca A, Puglisi MA, Serrano A, Marchese E, et al. The S100B story: from biomarker to active factor in neural injury. J Neurochem. 2019;148:168–87. 10.1111/jnc.14574. 30144068 10.1111/jnc.14574

[43] Bagchi S, Perland E, Hosseini K, Lundgren J, Al-Walai N, Kheder S, et al. Probable role for major facilitator superfamily domain containing 6 (MFSD6) in the brain during variable energy consumption. Int J Neurosci. 2020;130:476–89. 10.1080/00207454.2019.1694020. 31906755 10.1080/00207454.2019.1694020

[44] Ding L, Xu X, Li C, Wang Y, Xia X, Zheng JC. Glutaminase in microglia: a novel regulator of neuroinflammation. Brain Behav Immun. 2021;92:139–56. 33278560 10.1016/j.bbi.2020.11.038

[45] Han JS, Hino K, Li W, Reyes RV, Canales CP, Miltner AM, et al. CRL5-dependent regulation of the small GTPases ARL4C and ARF6 controls hippocampal morphogenesis. Proc Natl Acad Sci U S A. 2020;117:23073–84. 10.1073/pnas.2002749117. 32873638 10.1073/pnas.2002749117 PMC7502717

[46] Xu G, Wang R, Wang Z, Lei Q, Yu Z, Liu C, et al. NGL-2 is a new partner of PAR complex in axon differentiation. J Neurosci. 2015;35:7153–64. 10.1523/JNEUROSCI.4726-14.2015. 25948265 10.1523/JNEUROSCI.4726-14.2015 PMC6605266

[47] Wang J, Schreiber RD, Campbell IL. STAT1 deficiency unexpectedly and markedly exacerbates the pathophysiological actions of IFN-α in the central nervous system. Proc Natl Acad Sci U S A. 2002;99:16209–14. 10.1073/pnas.252454799. 12461178 10.1073/pnas.252454799 PMC138590

[48] Imitola J, Hollingsworth EW, Watanabe F, Olah M, Elyaman W, Starossom S, et al. Stat1 is an inducible transcriptional repressor of neural stem cells self-renewal program during neuroinflammation. Front Cell Neurosci. 2023;17:1156802. 10.3389/fncel.2023.1156802. 37663126 10.3389/fncel.2023.1156802 PMC10469489

[49] Yang Y, Hentati A, Deng H-X, Dabbagh O, Sasaki T, Hirano M, et al. The gene encoding alsin, a protein with three guanine-nucleotide exchange factor domains, is mutated in a form of recessive amyotrophic lateral sclerosis. Nat Genet. 2001;29:160–5. 10.1038/ng1001-160. 11586297 10.1038/ng1001-160

[50] Duranton M, Allal F, Valière S, Bouchez O, Bonhomme F, Gagnaire P-A. The contribution of ancient admixture to reproductive isolation between European sea bass lineages. Evol Lett. 2020;4:226–42. 10.1002/evl3.169. 32547783 10.1002/evl3.169 PMC7293100

[51] Duranton M, Bonhomme F, Gagnaire P-A. The spatial scale of dispersal revealed by admixture tracts. Evol Appl. 2019;12:1743–56. 31548854 10.1111/eva.12829 PMC6752141

[52] Duranton M, Allal F, Fraïsse C, Bierne N, Bonhomme F, Gagnaire P-A. The origin and remolding of genomic islands of differentiation in the European sea bass. Nat Commun. 2018;9:2518. 10.1038/s41467-018-04963-6. 29955054 10.1038/s41467-018-04963-6 PMC6023918

[53] Ebbesson LOE, Braithwaite VA. Environmental effects on fish neural plasticity and cognition. J Fish Biol. 2012;81:2151–74. 10.1111/j.1095-8649.2012.03486.x. 23252732 10.1111/j.1095-8649.2012.03486.x

[54] Le Verger K, Küng LC, Fabre A-C, Schmelzle T, Wegmann A, Sánchez-Villagra MR. Goldfish phenomics reveals commonalities and a lack of universality in the domestication process for ornamentation. Evol Lett. 2024;8(6):774–86. 10.1093/evlett/qrae032. 39677575 10.1093/evlett/qrae032 PMC11637523

[55] Mehlhorn J, Caspers S. The effects of domestication on the brain and behavior of the chicken in the light of evolution. Brain Behav Evol. 2021;95:287–301. 10.1159/000516787. 10.1159/000516787 34044402

[56] Li P, Chen P, Zheng Y, Suo G, Shen F, Li H, et al. Enhancement of motor neuron development and function in zebrafish by sialyllacto-N-tetraose b. Transl Pediatr. 2024;13:1201. 10.21037/tp-24-247. 39144427 10.21037/tp-24-247 PMC11319995

[57] van der Zee YY, Lardner CK, Parise EM, Mews P, Ramakrishnan A, Patel V, et al. Sex-specific role for *SLIT1* in regulating stress susceptibility. Biol Psychiatry. 2022;91:81–91. 33896623 10.1016/j.biopsych.2021.01.019 PMC8390577

[58] Xu Y, Li X, Zhong Y, Zheng Y. Evolution and diversity of axon guidance Robo receptor family genes. J Syst Evol. 2021;59:169–82. 10.1111/jse.12587.

[59] Li H, Chen J, Wu W, Fagaly T, Zhou L, Yuan W, et al. Vertebrate slit, a secreted ligand for the transmembrane protein roundabout, is a repellent for olfactory bulb axons. Cell. 1999;96:807–18. 10.1016/s0092-8674(00)80591-7. 10102269 10.1016/s0092-8674(00)80591-7

[60] Telese F, Ma Q, Perez PM, Notani D, Oh S, Li W, et al. LRP8-Reelin-regulated neuronal enhancer signature underlying learning and memory formation. Neuron. 2015;86:696–710. 25892301 10.1016/j.neuron.2015.03.033 PMC4486257

[61] Fujita E, Tanabe Y, Imhof BA, Momoi MY, Momoi T. A complex of synaptic adhesion molecule CADM 1, a molecule related to autism spectrum disorder, with MUPP 1 in the cerebellum. J Neurochem. 2012;123:886–94. 10.1111/jnc.12022. 22994563 10.1111/jnc.12022

[62] O’Leary A, Fernàndez-Castillo N, Gan G, Yang Y, Yotova AY, Kranz TM, et al. Behavioural and functional evidence revealing the role of RBFOX1 variation in multiple psychiatric disorders and traits. Mol Psychiatry. 2022;27:4464–73. 10.1038/s41380-022-01722-4. 35948661 10.1038/s41380-022-01722-4 PMC9734045

[63] Vaz R, Edwards S, Dueñas-Rey A, Hofmeister W, Lindstrand A. Loss of ctnnd2b affects neuronal differentiation and behavior in zebrafish. Front Neurosci. 2023;17:1205653. 10.3389/fnins.2023.1205653. 37465584 10.3389/fnins.2023.1205653 PMC10351287

[64] Matsui A, Tran M, Yoshida AC, Kikuchi SS, U M, Ogawa M, et al. BTBD3 controls dendrite orientation toward active axons in mammalian neocortex. Science. 2013;342:1114–8. 10.1126/science.1244505. 24179155 10.1126/science.1244505

[65] Araki M, Nagata K, Satoh Y, Kadota Y, Hisha H, Adachi Y, et al. Developmentally regulated expression of Neuro-p24 and its possible function in neurite extension. Neurosci Res. 2002;44:379–89. 12445626 10.1016/s0168-0102(02)00156-6

[66] Yang J, Kong L, Zou L, Liu Y. The role of synaptic protein NSF in the development and progression of neurological diseases. Front Neurosci. 2024;18:1395294. 10.3389/fnins.2024.1395294. 39498393 10.3389/fnins.2024.1395294 PMC11532144

[67] Temel RE, Tang W, Ma Y, Rudel LL, Willingham MC, Ioannou YA, et al. Hepatic Niemann-Pick C1–like 1 regulates biliary cholesterol concentration and is a target of ezetimibe. J Clin Invest. 2007;117:1968–78. 10.1172/JCI30060. 17571164 10.1172/JCI30060 PMC1888567

[68] Hu Y, Zhang M, Sun L. Expression of *Scophthalmus maximus* CD83 correlates with bacterial infection and antigen stimulation. Fish Shellfish Immunol. 2010;29:608–14. 20561589 10.1016/j.fsi.2010.06.014

[69] Soleto I, Fischer U, Tafalla C, Granja AG. Identification of a potential common ancestor for mammalian cross-presenting dendritic cells in teleost respiratory surfaces. Front Immunol. 2018;9:59. 10.3389/fimmu.2018.00059. 29422901 10.3389/fimmu.2018.00059 PMC5788898

[70] Müller P-S, Schulz R, Maretto S, Costello I, Srinivas S, Bikoff E, et al. The fibronectin leucine-rich repeat transmembrane protein Flrt2 is required in the epicardium to promote heart morphogenesis. Development. 2011;138:1297–308. 10.1242/dev.059386. 21350012 10.1242/dev.059386 PMC3050662

[71] Errichiello E, Lecca M, Vantaggiato C, Motta Z, Zanotta N, Zucca C, et al. Further evidence supporting the role of GTDC1 in glycine metabolism and neurodevelopmental disorders. Eur J Hum Genet. 2024;32:920–7. 10.1038/s41431-024-01603-0. 38605125 10.1038/s41431-024-01603-0 PMC11291697

[72] Nguyen LTM, Hassan S, Pan H, Wu S, Wen Z. Interplay of Zeb2a, Id2a and Batf3 regulates microglia and dendritic cell development in the zebrafish brain. Development. 2024;151:dev201829. 10.1242/dev.201829. 38240311 10.1242/dev.201829

[73] Kito K, Yeh ETH, Kamitani T. NUB1, a NEDD8-interacting protein, is induced by interferon and down-regulates the NEDD8 expression*. J Biol Chem. 2001;276:20603–9. 11259415 10.1074/jbc.M100920200

[74] Pavelin J, Jin YH, Gratacap RL, Taggart JB, Hamilton A, Verner-Jeffreys DW, et al. The nedd-8 activating enzyme gene underlies genetic resistance to infectious pancreatic necrosis virus in Atlantic salmon. Genomics. 2021;113:3842–50. 34547402 10.1016/j.ygeno.2021.09.012 PMC8682971

[75] Gundappa MK, Robledo D, Hamilton A, Houston RD, Prendergast JGD, Macqueen DJ. High performance imputation of structural and single nucleotide variants using low-coverage whole genome sequencing. Genet Sel Evol. 2025;57:16. 10.1186/s12711-025-00962-6. 40155798 10.1186/s12711-025-00962-6 PMC11951665

[76] Anastasiadi D, Piferrer F. Epimutations in developmental genes underlie the onset of domestication in farmed European Sea bass. Mol Biol Evol. 2019;36:2252–64. 10.1093/molbev/msz153. 31289822 10.1093/molbev/msz153 PMC6759067

[77] Pedersen BS, Quinlan AR. Mosdepth: quick coverage calculation for genomes and exomes. Bioinformatics. 2018;34:867–8. 10.1093/bioinformatics/btx699. 29096012 10.1093/bioinformatics/btx699 PMC6030888

[78] Bolger AM, Lohse M, Usadel B. Trimmomatic: a flexible trimmer for Illumina sequence data. Bioinformatics. 2014;30:2114–20. 10.1093/bioinformatics/btu170. 24695404 10.1093/bioinformatics/btu170 PMC4103590

[79] Li H, Durbin R. Fast and accurate short read alignment with Burrows-Wheeler transform. Bioinformatics. 2009;25:1754–60. 10.1093/bioinformatics/btp324. 19451168 10.1093/bioinformatics/btp324 PMC2705234

[80] Li H, Handsaker B, Wysoker A, Fennell T, Ruan J, Homer N, et al. The sequence alignment/map format and SAMtools. Bioinformatics. 2009;25:2078–9. 10.1093/bioinformatics/btp352. 19505943 10.1093/bioinformatics/btp352 PMC2723002

[81] Layer RM, Chiang C, Quinlan AR, Hall IM. LUMPY: A probabilistic framework for structural variant discovery. Genome Biol. 2014;15:1–19. 10.1186/gb-2014-15-6-r84. 10.1186/gb-2014-15-6-r84 PMC4197822 24970577

[82] Chiang C, Layer RM, Faust GG, Lindberg MR, Rose DB, Garrison EP, et al. Speedseq: ultra-fast personal genome analysis and interpretation. Nat Methods. 2015;12:966–8. 10.1038/nmeth.3505. 26258291 10.1038/nmeth.3505 PMC4589466

[83] Pedersen BS, Quinlan AR. Duphold: scalable, depth-based annotation and curation of high-confidence structural variant calls. Gigascience. 2019;8:giz040. 10.1093/gigascience/giz040. 31222198 10.1093/gigascience/giz040 PMC6479422

[84] Belyeu JR, Nicholas TJ, Pedersen BS, Sasani TA, Havrilla JM, Kravitz SN, et al. SV-plaudit: A cloud-based framework for manually curating thousands of structural variants. Gigascience. 2018;7:1–7. 10.1093/gigascience/giy064. 10.1093/gigascience/giy064 PMC6030999 29860504

[85] Belyeu JR, Chowdhury M, Brown J, Pedersen BS, Cormier MJ, Quinlan AR, et al. Samplot: a platform for structural variant visual validation and automated filtering. Genome Biol. 2021;22:1–13. 10.1186/s13059-021-02380-5. 34034781 10.1186/s13059-021-02380-5 PMC8145817

[86] Danecek P, Auton A, Abecasis G, Albers CA, Banks E, DePristo MA, et al. The variant call format and VCFtools. Bioinformatics. 2011;27:2156–8. 10.1093/bioinformatics/btr330. 21653522 10.1093/bioinformatics/btr330 PMC3137218

[87] Cingolani P, Platts A, Wang LL, Coon M, Nguyen T, Wang L, et al. A program for annotating and predicting the effects of single nucleotide polymorphisms, SnpEff: SNPs in the genome of *Drosophila melanogaster* strain w1118; iso-2; iso-3. Fly. 2012;6:80–92. 10.4161/fly.19695. 22728672 10.4161/fly.19695 PMC3679285

[88] Harrison PW, Amode MR, Austine-Orimoloye O, Azov AG, Barba M, Barnes I, et al. Ensembl 2024. Nucleic Acids Res. 2024;52:D891–9. 10.1093/nar/gkad1049. 37953337 10.1093/nar/gkad1049 PMC10767893

[89] Flynn JM, Hubley R, Goubert C, Rosen J, Clark AG, Feschotte C, et al. Repeatmodeler2 for automated genomic discovery of transposable element families. Proc Natl Acad Sci U S A. 2020;117:9451–7. 10.1073/pnas.1921046117. 32300014 10.1073/pnas.1921046117 PMC7196820

[90] Quinlan AR, Hall IM. BEDtools: a flexible suite of utilities for comparing genomic features. Bioinformatics. 2010;26:841–2. 10.1093/bioinformatics/btq033. 20110278 10.1093/bioinformatics/btq033 PMC2832824

[91] Herrero J, Muffato M, Beal K, Fitzgerald S, Gordon L, Pignatelli M, et al. Ensembl comparative genomics resources. Database. 2016;2016:bav096. 10.1093/database/baw053. 26896847 10.1093/database/bav096 PMC4761110

[92] Paysan-Lafosse T, Blum M, Chuguransky S, Grego T, Pinto BL, Salazar GA, et al. InterPro in 2022. Nucleic Acids Res. 2023;51:D418–27. 10.1093/nar/gkac993. 36350672 10.1093/nar/gkac993 PMC9825450

[93] Purcell S, Neale B, Todd-Brown K, Thomas L, Ferreira MAR, Bender D, et al. PLINK: a tool set for whole-genome association and population-based linkage analyses. Am J Hum Genet. 2007;81:559–75. 10.1086/519795. 17701901 10.1086/519795 PMC1950838

[94] Liao Y, Smyth GK, Shi W. Featurecounts: an efficient general purpose program for assigning sequence reads to genomic features. Bioinformatics. 2014;30:923–30. 10.1093/bioinformatics/btt656. 24227677 10.1093/bioinformatics/btt656

[95] Huerta-Cepas J, Forslund K, Coelho LP, Szklarczyk D, Jensen LJ, Von Mering C, et al. Fast genome-wide functional annotation through orthology assignment by eggNOG-mapper. Mol Biol Evol. 2017;34:2115–22. 10.1093/molbev/msx148. 28460117 10.1093/molbev/msx148 PMC5850834

[96] Yu G, Wang L-G, Han Y, He Q-Y. Clusterprofiler: an R package for comparing biological themes among gene clusters. OMICS. 2012;16:284–7. 10.1089/omi.2011.0118. 22455463 10.1089/omi.2011.0118 PMC3339379

[97] Wickham H. ggplot2: Elegant Graphics for Data Analysis. springer New York; 2016. 10.1007/978-3-319-24277-4.

[98] Yin L, Zhang H, Tang Z, Xu J, Yin D, Zhang Z, et al. RMVP: a memory-efficient, visualization-enhanced, and parallel-accelerated tool for genome-wide association study. Genomics Proteomics Bioinformatics. 2021;19:619–28. 10.1016/j.gpb.2020.10.007. 33662620 10.1016/j.gpb.2020.10.007 PMC9040015

[99] Conway JR, Lex A, Gehlenborg N. UpsetR: an R package for the visualization of intersecting sets and their properties. Bioinformatics. 2017;33:2938–40. 10.1093/bioinformatics/btx364. 28645171 10.1093/bioinformatics/btx364 PMC5870712

[100] Kolde R, Kolde MR. Package ‘pheatmap.’ R Packag. 2015;1:790.

